# PRDX1 promotes clear cell renal cell carcinoma progression by modulating EGFR-dependent AKT pathway activation

**DOI:** 10.3389/fphar.2026.1741879

**Published:** 2026-04-17

**Authors:** Zhi Liang, Xiaochun Lin, Shen Zeng, Lili Wu, Yujun Dong, Chen Zhang, Mei Li, Fudi Zhu, Lu Chen, Suiqin Ni

**Affiliations:** 1 Department of Pharmacy, Guangzhou First People’s Hospital, South China University of Technology, Guangzhou, Guangdong, China; 2 Department of Pharmacy, The First People’s Hospital of Foshan (Foshan Hospital Affiliated to Southern University of Science and Technology), School of Medicine, Southern University of Science and Technology, Foshan, Guangdong, China; 3 Office of the Ethics Committee of Guangzhou First People’s Hospital, Southern University of Science and Technology, Guangzhou, Guangdong, China

**Keywords:** clear cell renal cell carcinoma, clinical prognosis, EGFR signaling pathway, peroxiredoxin 1, survival rate

## Abstract

**Background:**

Renal cell carcinoma (RCC) is a highly heterogeneous malignant tumor, characterized by a globally increasing incidence and mortality rate. Although surgical resection serves as the standard treatment for localized RCC, recurrence and metastasis remain major clinical challenges. Based on patient sample analysis and signaling pathway investigation, the present study identifies peroxiredoxin 1 (PRDX1) as a potential therapeutic target for RCC.

**Methods:**

Patient microarray analysis, combined with data from The Cancer Genome Atlas (TCGA) and Gene Expression Omnibus (GEO) databases, revealed the expression pattern of PRDX1 in clear cell renal cell carcinoma (ccRCC) and confirmed its prognostic and diagnostic significance. Cell proliferation was evaluated using Cell Counting Kit-8 (CCK-8) assay, while cell motility was assessed via wound healing and transwell assays. Gene expression profiles and signaling pathways were analyzed through bioinformatics approaches including Western blot, immunohistochemistry and immunoprecipitation. A xenograft mouse model was utilized to investigate the *in vivo* effects of PRDX1.

**Results:**

The analysis of 90 pairs of ccRCC samples demonstrated that elevated PRDX1 expression was significantly associated with higher malignancy in ccRCC and correlated with tumor prognosis and clinical stage. Comprehensive bioinformatics analysis identified a close relationship between PRDX1 inhibition and relative epidermal growth factor receptor (EGFR) pathways. Mechanistically, co-immunoprecipitation (Co-IP) assays revealed that PRDX1 could act as a molecular chaperone by binding to the juxta-membrane (JM) domain of EGFR to induce EGFR phosphorylation. The downregulation of PRDX1 significantly reduced their proliferation and migration capacities in renal cell lines ACHN and 786-O, whereas PRDX1 overexpression exerted the opposite effect. Importantly, the inhibitory effects of PRDX1 knockdown on ccRCC could be attenuated by EGF activation *in vitro*, as well as the oncogenic functions enhanced by increasing PRDX1 was blocked by gefitinib, a specific inhibitor of EGFR. Treatment with PRDX1 inhibitor showed that PRDX1 inhibition restrain the growth and metastasis of ccRCC.

**Conclusion:**

In summary, the findings of this study revealed a novel role of PRDX1 in triggering ccRCC progression by inducing the phosphorylation of EGFR, supporting that PRDX1 may serve as a potential therapeutic target for the clinical management of ccRCC.

## 1 Introduction

The incidence rate of kidney cancer is increasing by 3% annually, and it has become one of the most common malignant tumors in the urinary system, second only to bladder cancer (Rose and Kim, 2024). According to *The Cancer Atlas, 4th edition* from the International Agency for Research on Cancer (IARC), kidney cancer caused more than 165,000 deaths globally (Knol et al., 2025). Renal cell carcinoma (RCC) is a solid malignant tumor originating from renal epithelial cells, the most common form of kidney cancer. Due to the difficulty in detecting early symptoms of renal cell carcinoma, some patients are diagnosed at an advanced stage (Bex et al., 2025). There are two groups of patients with a particularly high risk of dying from renal cell carcinoma: those with metastatic disease and those with postoperative recurrence. Approximately 30% of renal cell carcinoma patients initially present with metastatic disease. Meanwhile, about 30% of patients who underwent surgery for complete tumor removal experienced local recurrence, leading to a poor prognosis and a failure to extend the overall survival time of patients. Therefore, advanced renal cell carcinoma is a fatal disease, with a 5 year survival rate of only 11.7% (Motzer et al., 2024). The clear cell renal cell carcinomas (ccRCCs) represent nearly 70%–80% of RCC, and account for most RCC-associated deaths.

Peroxiredoxins (PRDXs), a group of unique proteins, exhibit high cellular abundance and are present in all known species and most cellular compartments, including the cytoplasm, nucleus, mitochondria, and peroxisomes (Hall et al., 2009). As a member of the PRDX family, PRDX1 was found the highest abundance and serve as an antioxidant enzyme. In addition to its initial role as an antioxidant to protect cells from oxidative stress damage, PRDX1 is also involved in the regulation of apoptosis, progression of other intracellular processes such as cell cycle, DNA damage responses, and anti-tumor immunity (Ahmed and Lingner, 2020; Hinshaw and Shevde, 2019; Ledgerwood et al., 2017; Li et al., 2025). The relationship between its subcellular localization and different functions has raised the attention of researchers. After decades of research, how PRDX1 traffics among these locations and performs different functions has been partially elucidated. According to its binding proteins, the molecular chaperone activity of PRDX1 was gradually discovered. For instance, Song et al. found that PRDX1 inhibits ferroptosis by binding to Cullin-3 as a molecular chaperone in colorectal cancer (CRC) (Song et al., 2024). Yoon et al. revealed that PRDX1 directly interacts with the ring finger domain of TRAF6, negatively regulating NFKB activation and autophagy functions (Hansen et al., 2007).

Tumor cells often display an extremely active metabolism, leading to the abnormal accumulation of reactive oxygen species (ROS). As the key antioxidant enzyme, PRDX1 exhibits high expression levels in most human solid tumors, such as triple-negative breast cancer (Feng et al., 2025; Spinola-Lasso et al., 2023), non-small cell lung cancer (NSCLC) (Hao et al., 2023; Wang et al., 2025), hepatocellular carcinoma (HCC) (Meng et al., 2025; Yang et al., 2025), ovarian cancer (OC) (Shi et al., 2025; Fan et al., 2024), and cervical cancer (He et al., 2025; Sun et al., 2024), suggesting the possible role of PRDX1 in oncogenesis. However, the role in renal cell carcinoma still remains unclear.

Previous studies revealed that targeting PRDX1 was an effective strategy in the preclinical setting for multiple types of cancers, indicating the promising therapeutic potential of anti-PRDX1 therapy (Guan et al., 2024). PRDX1-targeted drugs have demonstrated anti-cancer effects *in vitro*, indicating the potential of PRDX1 as a molecular target. The aim of this study is to assess the value of PRDX1 in renal cell carcinoma treatment using kidney cancer cell lines, *in vivo* xenograft models and tissue microarrays (TMAs) of human ccRCC. We will discuss the role of PRDX1 in tumor cell behaviors, aiming to offer potential insights for new tumor treatment regimens and to establish a foundation for further comprehensive investigations of PRDX1-mediated apoptosis that may benefit clinical patients. We propose that future works on PRDX1 inhibitors may act as a therapeutic candidate for treatment of renal cancer.

## 2 Materials and methods

### 2.1 Tissue microarrays (TMAs) and ethics

Tissue microarrays (TMAs) of human ccRCC, containing 90 tumor samples and 90 adjacent normal tissue samples in pairs, were obtained from OUTDO Biotech (HKidE180Su03; Shanghai, China). The immunohistochemistry (IHC) scores were calculated based on the percentage of positively-stained cells and staining intensity, as previously described (Chen et al., 2019). Detailed information can be found at the following website: https://www.superchip.com.cn/.

### 2.2 Data collection

The UALCAN database (https://portal.gdc.cancer.gov/) was used to estimate the gene transcription, protein expression, clinical features of PRDX1 in patients with ccRCC. Datasets GSE40435 and GSE73731 were downloaded from the NCI/NCBI GEO database (https://www.ncbi.nlm.nih.gov/geo/). Co-expression network construction and functional prediction analysis were conducted using the GSE40435 dataset. A comparative analysis was performed between normal kidney and ccRCC tissues using the TCGA-KIRC, GSE40435, and GSE53757 datasets. All data analyses were conducted using R (version 4.1.2) and R Bioconductor packages (Yu et al., 2012). The obtained reads (counts) were normalized to their library sizes and transcript length (TPM normalization). To compare the percentage survival between different PRDX1 expression groups, Kaplan–Meier survival curves were generated. Related hub genes and pathways were acquired by Kyoto Encyclopedia of Genes and Genomes (KEGG) and Gene Set Enrichment Analysis (GSEA) (Yang et al., 2022).

### 2.3 Cell culture and reagents

Human ACHN, 786-O, 293T cells were provided by the Chinese Academy of Sciences in Shanghai. ACHN cells were cultured using the MEM (Gibco, Uncited States),786-O cells were cultured using the 1,640 medium (Gibco, Uncited States), and 293T cells were cultured using the DMEM (High glucose) (Gibco, Uncited States). All cell cultures were grown at 37 °C in a humidified incubator containing 5% CO_2_ with 10% fetal bovine serum (FBS) (Gibco, Thermo Fisher Scientific, Uncited States). The drugs and chemicals used in this study, including PRDX1 inhibitor (PRDX1-IN-1), epidermal growth factor (EGF), were purchased from MCE (Shanghai, China). Cells were treated with 100 ng/mL epidermal growth factor (EGF) (MCE, Shanghai, China) for 30 min. EGFR (4267S, CST), p-EGFR (Tyr1068, 3777S, CST), AKT (4691T,CST), p-AKT (Ser473, 4060L, CST), PI3K (4257T, CST), p-PI3KP85(Tyr458, 4228S, CST), Snail (3879S, CST), E-cad (3195S, CST), N-cad (13116S, CST), KI67 (ab15580, Abcam) and β-Actin (sc-47778, Santa Cruz) were purchased from indicated commercial sources. Matrigel Matrix (Corning, Uncited States) was used in transwell assay.

### 2.4 Plasmid and RNA interference

The gene overexpressing plasmid used for PRDX1 was purchased from OBiO Technology (Shanghai) Corp., Ltd and GENEWIZ Suzhou Co., Ltd. PRDX1 knockout cells were created using the CRISPR-Cas9 system with single guide RNAs (sgRNAs see in Supplementary Table S1) designed by the Cyagen Biosciences (Suzhou, China). The full-length EGFR and the corresponding truncated plasmids were subcloned into a pIRES2-EGFP-Myc vector. Two small interfering RNAs (siRNAs) targeting PRDX1 (PRDX1-siRNA1, PRDX1-siRNA2) were used to silence the expression of PRDX1 (See in Supplementary Table S1). The lentivirus expressing short hairpin RNA (shRNA) targeting PRDX1 was also constructed and packaged by Tsingke Biotechnology Co., Ltd (Beijing, China). Lipofectamine 3,000 (Invitrogen, Carlsbad, CA, Uncited States) was used for cell transfection according to the manufacturer’s instructions.

### 2.5 Quantitative real-time PCR

RNA extraction was typically performed using RNeasy Plus Mini Kits (Qiagen, Germany) according to the manufacturer’s instructions. After extraction, RNA quality and concentrations were assessed through a spectrophotometer. Then the mRNA levels were detected by SYBR Green real-time PCR Master Mix (Vazyme Biotech Co., Ltd) and performed on ABI QuantStudio 5 Real-Time PCR System. Supplementary Table S1 included primer sequences for the experiments. The mRNA expression was normalized by the gene expression levels to β-actin, which was used as an internal control.

### 2.6 Cell proliferation assay

Four assays were used to assess cell proliferation: the colony formation assay, the wound healing assay, the transwell cell invasion assay and the cell counting kit-8 (CCK-8) assay. The colony formation assay: In 6-well plates, 800 cells were seeded per well for 10–14 days. Colonies were counted after staining with 1% crystal violet for 10 min. The wound healing assay: In 6-well plates, 10 × 10^5^ cells were seeded per well for 24 h. Then a 200 µL pipette tip was used to scratch the monolayer of confluent cells in the center and along the entirety of the well’s diameter. After washing gently with PBS, cells were obtaining images within 0–12 h. The transwell invasion assay: Cells were counted and diluted to a density of 5 × 10^5^/mL with serum-free medium. Next, 150 μL of the cell suspension was added to the upper compartment with an 8-μm pore size membrane (BD Biosciences, Uncited States) (for the invasion assay), which was cultured with diluted Matrigel solution at 37 °C for 1 h prior to starting the experimental protocol. The final cell density was 7.5 × 10^4^ cells/well. Carefully add 700 μL of the cultured medium with 10% FBS to the well directly below the 24-well transwell insert. Incubate the plate at 37 °C and 5% CO_2_ for 16–24 h. As a control, 700 μL medium without FBS was added to the lower chambers. The cell counting kit-8 (CCK-8) assay: cells were seeded into 96-well plates at a concentration of 5 × 10^3^/well with 100 μL culture medium. Until the cells were cultured accordingly, 10 μL CCK-8 reagent was added to each well. The absorbance at 450 nm was detected after an incubation period of 2–4 h.

### 2.7 Immunofluorescence assay

Cells were prepared for cell staining by growing on a suitable plastic tissue culture dish. After washing with PBS, cells were fixed in 4% PFA at room temperature for 30 min and then cells were incubated with the appropriate antibodies overnight at 4 °C after another 3 times washing with PBS. Fluorescein-labeled secondary antibodies were added and incubated for 2 h at room temperature after three additional PBS washes. DAPI (D8417, Sigma-Aldrich, Germany) was incubated with cells for 15–30 min at room temperature followed by mounting with mounting medium. A laser scanning confocal microscope (LSM900, Zeiss, Germany) was employed to analyze the slides.

### 2.8 Co-immunoprecipitation

Cells were transfected with different plasmids for 48–72 h as previously described (Zhang et al., 2023). After washing by cold PBS for 3 times, suspended cells were lysed with 400 μL 1 x IP lysis buffer (9803S, CST) for 30 min on ice. After centrifugating for 15 min at 12,000x g in a cold microfuge, the supernatant was removed for use. Briefly, approximately 200 μg of total cellular proteins was incubated with the target antibody overnight at 4 °C. Protein A/G PLUS-agarose beads (sc-2003, Santa Cruz) were added, and control IgG (Abclonal, Wuhan, China) was included in the reaction as a negative control.

### 2.9 *In vivo* xenograft model

The animal experiment protocol was approved by the Ethics Committee of South China University of Technology, China. Male BALB/c nude mice aged 4 weeks were kept in a specific pathogen-free (SPF) environment and acclimatized for 7 days. Transfected ACHN cells were resuspended in cold PBS buffer mixed with Matrigel. A mixture containing 1 × 10^7^ cells was subcutaneously injected into the axilla of each mouse. The tumor volume was measured and calculated using the formula: tumor volume = (length × width^2^)/2 in mm^3^. All the mice were humanely euthanized to obtain fresh tumors and measure tumor weight. The subcutaneous tumor specimens were collected for further analysis through hematoxylin and immunohistochemical staining.

### 2.10 Statistics

Data were presented as mean ± SD of independent experiments. The clinical pathologic information (Supplementary Table S2) was analyzed using Wilcoxon’s test. Univariate and multivariate Cox proportional-hazard models were used to identify independent prognostic factors for ccRCC patients (Kaulen et al., 2024). GraphPad Prism 9 (GraphPad Software, Uncited States) was utilized for statistical analysis. A P-value less than 0.05 was considered statistically significant. Actual P-values for significance presented in the figures was listed in Supplementary Table S3. Statistical significance was denoted by ‘****’ = P < 0.0001, ‘***’ = P < 0.001, ‘**’ = P < 0.01, ‘*’ = P < 0.05.

## 3 Results

### 3.1 PRDX1 expression is strongly associated with the malignancy of ccRCC

**FIGURE 1:**
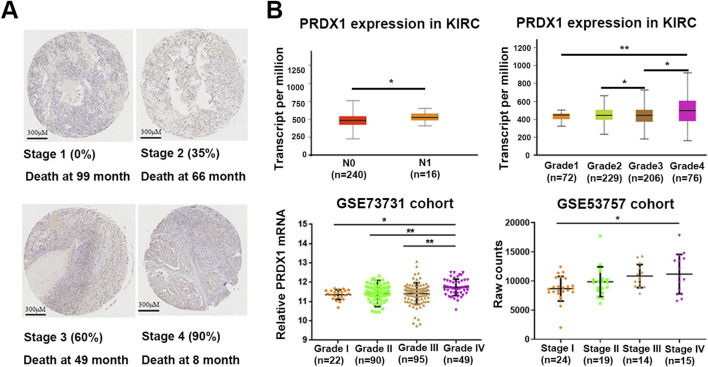
The expression data of PRDX1 obtained from tissue microarray (TMA) of human ccRCC samples and UALCAN database. **(A)** Representative PRDX1 IHC images of ccRCC tissues with overall survival. Scale bar: 300 μm. **(B)** PRDX1 expression was highly associated with higher tumor stage and lymphatic metastasis in ccRCC. Statistical significance was assessed using unpaired student’s t-tests.

To explore the role of PRDX1 in ccRCC progression, we performed IHC staining on 90 pairs of ccRCC tissue microarrays (TMAs) to assess the expression of PRDX1. As shown in Figure 1A, PRDX1 expression in patients with early-stage renal cancer was significantly lower than in those with advanced-stage disease, concomitant with longer survival lifespan. Additional evidence supporting these findings was obtained from transcriptome data of UALCAN database and GEO database including GSE73731 and GSE53757 cohorts (Figure 1B). Besides, univariate and multivariate Cox proportional models were used to identify independent prognostic factors for ccRCC patients. Age, extent of lymph node metastases (N), occurrence of distant metastases (M), grade and the TNM system were found to be independent risk factors for tumor progression (Supplementary Figures S1, S2). Taken together, these results indicated that the expression of PRDX1 was strongly associated with the malignancy and survival of renal cell carcinoma.

### 3.2 Knockdown of PRDX1 inhibits ccRCC aggression *in vitro*

**FIGURE 2:**
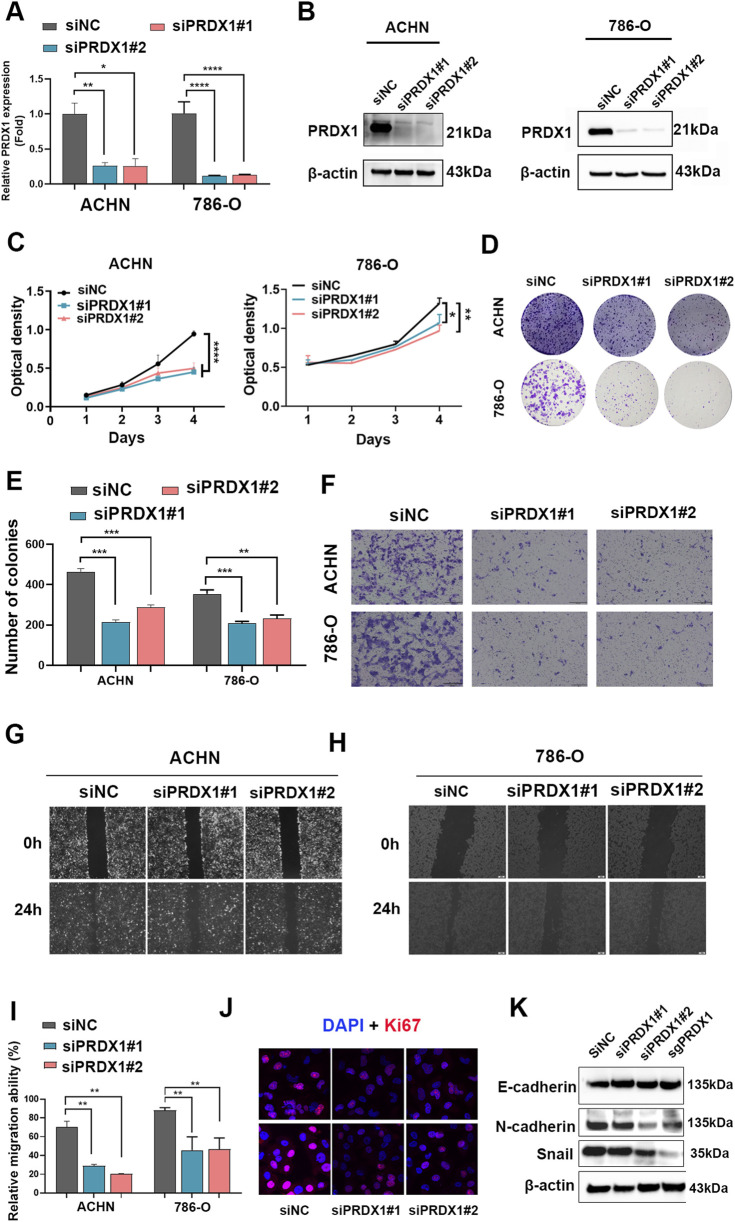
The variations of malignant biological function in PRDX1-downregulating ccRCC cell lines. The interference efficiency of PRDX1-silent cell lines was evaluated by **(A)** RT-qPCR and **(B)** immunoblot analysis. **(C)** CCK8 assay revealed that PRDX1 downregulation attenuated cell viability in ACHN and 786-O cells. PRDX1 downregulation attenuated **(D,E)** clone formation, **(F)** cell invasion as well as **(G–I)** cell migration viability in ACHN and 786-O cells. **(J)** Immunofluorescence assay examined the effect of PRDX1 downregulation on Ki-67 expression in ACHN and 786-O cells. Scale bar = 200 μm. **(K)** Immunoblot assay verified the EMT-related proteins’ expression in ACHN cells. Data were presented as mean ± SD of 3 independent experiments and assessed using *t*-tests.

To examine the specific role of PRDX1 in ccRCC, we inhibited PRDX1 in ACHN and 786-O cells through siRNA transfection (Figures 2A,B). As shown in Figure 2C, CCK-8 assay revealed that the inhibition of PRDX1 greatly reduced the viability of cell proliferation. Furthermore, PRDX1 inhibition significantly decreased the migratory capabilities and invasive ability of ccRCC cells using cell scratch test and transwell assay (Figures 2D–I). The nuclear protein Ki67 (pKi67) is a prognostic and predictive indicator for the assessment of biopsies from patients with cancer. As shown in Figure 2J, the Ki67-positive cells were sharply induced when PRDX1 was knocked down. In addition, epithelial mesenchymal transition (EMT) is considered a cellular program that is known to be crucial for tumor malignant progression. We further detected the EMT markers (E-cadherin, N- cadherin, and Snail) via immunoblot analysis. Inhibition of PRDX1 upregulated E-cadherin expression while downregulating N-cadherin and Snail levels (Figure 2K). This suggests that PRDX1 may promote the malignant characteristics of ccRCC cells.

### 3.3 Silencing PRDX1 suppressed tumor growth *in vivo*

**FIGURE 3:**
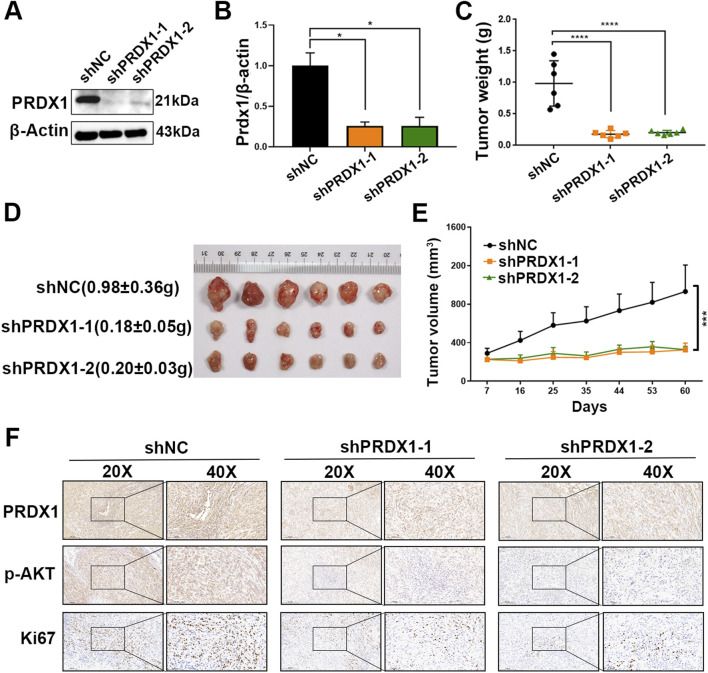
Inhibition of PRDX1 suppresses tumor growth *in vivo*. The interference efficiency of shPRDX1 was evaluated by **(A)** RT-qPCR and **(B)** immunoblot analysis. PRDX1 inhibition markedly reduced **(C)** the weight and **(E)** volume of the tumor xenograft as well as inhibited **(D)** tumor growth (n = 6 in each group). **(F)** Representative IHC staining of tumor tissues showed the expression of PRDX1, p-AKT and Ki67. Statistical significance was assessed using One-way ANOVA with Dunnett’s multiple comparisons post-test.

Further exploration was conducted in the xenograft mouse model to determine whether PRDX1 contributes to tumor growth. Two sequence-specific shRNA1 and shRNA2 were designed to silent the expression of PRDX1 in ACHN cell detecting by qPCR and Western blotting (Figures 3A,B). Xenograft mice models were generated by subcutaneous implantation of ACHN control cells (shNC), and PRDX1-silent ACHN cells (shPRDX1-1 & shPRDX1-2) into BALB/c nude mice. As shown in Figures 3C–E, silencing PRDX1 significantly repressed the weights and volumes of tumors. Moreover, IHC assay demonstrated that the shPRDX1 groups showed lower positive p-AKT and Ki67 staining than the NC group (Figure 3F). These findings supported that the inhibition of PRDX1 blocked the tumor growth *in vivo*.

### 3.4 Knockdown of PRDX1 attenuating EGFR/PI3K signaling axis

**FIGURE 4:**
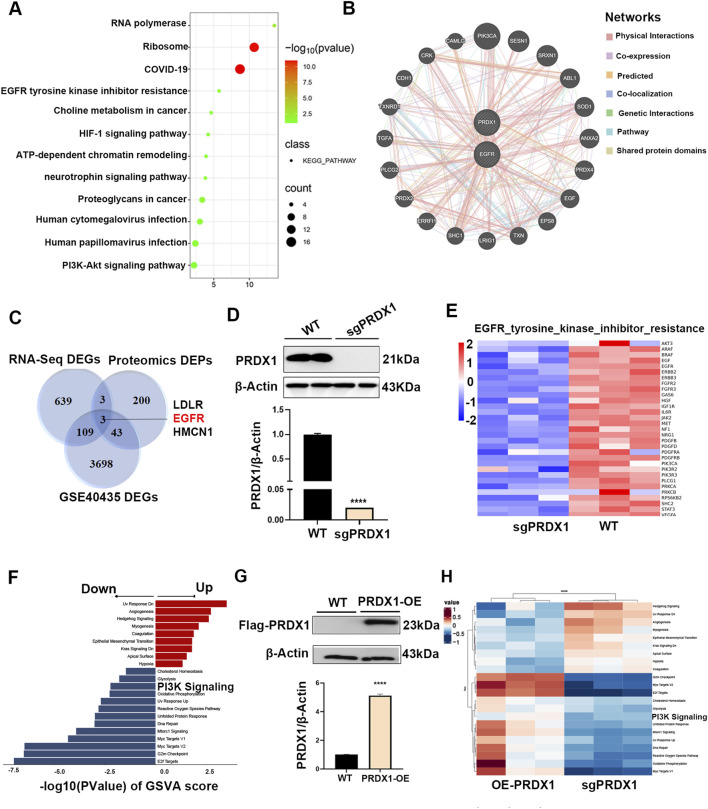
PRDX1 attenuates EGFR/PI3K signaling axis. **(A)** KEGG pathway enrichment analysis and **(B)** the supervised weighted correlation network analysis of GSE116158. **(C)** Intersection of DEGs and DEPs affected by PA as well as those DEGs from GSE40435. **(D)** The protein levels of PRDX1 in ACHN cells with sgPRDX1 transfection (n = 3 for independent biological replicates). **(E)** Heatmap plot showed the relative EGFR pathway hub genes were negatively related with PRDX1-knockout in ACHN cell. **(F)** GSVA result indicated PI3K pathway was significantly downregulated in PRDX1-knockout ACHN cell. **(G)** The protein levels of PRDX1 in ACHN cells with overexpressing PRDX1 transfection (n = 3 for independent biological replicates). **(H)** Heatmap plot indicated that PI3K signaling was activated in aberrant PRDX1 expressing ACHN cell. All bioinformatics analysis was based on 3 independent biological replicates.

In order to elucidate the molecular mechanism by which PRDX1 affects the oncogenic functions of renal cell carcinoma, we analyzed the proteomics data of ACHN cell exposed with a PRDX1 regulator, piericidin A (PA), as previously reported (GSE116158). Screening was performed (Log2|Fc|≥1, P < 0.05), and totally 249 statistically significant differentially expression proteins (DEPs) were obtained. KEGG pathway enrichment analysis was employed to indicated the biological characterization of the significantly regulated proteins (Figure 4A). The supervised weighted correlation network analysis (sWGCNA) was employed to define hub proteins and the relationship among the selected DEPs (Langfelder and Horvath, 2008) (Figure 4B). When the nodes are larger and darker, the degree of the node is larger. As a result, 22 hub proteins were highly correlated, while PRDX1 and EGFR shared the largest nodes, suggesting their biological function might be related. Additionally, Venn software (https://bioinfogp.cnb.csic.es/tools/venny/index.html) was employed to analyze the overlap between disease targets obtained from GSE40435 and drug targets obtained by RNA-seq and proteomics treated with PA (Figure 4C). Furthermore, we constructed PRDX1-depleted ACHN cells (sgPRDX1) as well as PRDX1-overexpressing ACHN cells (OE-PRDX1) to substantiate this finding (Figures 4D,G). As shown in Figure 4E, the relative genes of EGFR tyrosine kinase inhibitor resistance pathway were differentially downregulated in PRDX1-koockout cells, which also verified by GSVA score (Figure 4F). Similarly, when compared with PRDX1-overexpressing cells, PI3K signaling pathway was also downregulated in sgPRDX1 cells (Figure 4H). It could be concluded that there was a close association between PRDX1 and EGFR pathway.

### 3.5 PRDX1 interacts with the EGF receptor (EGFR)

**FIGURE 5:**
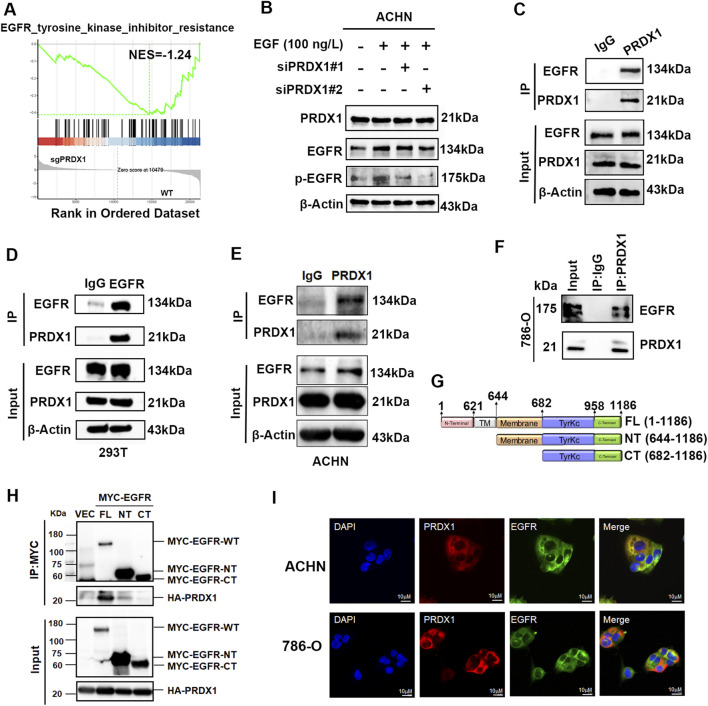
PRDX1 interacts with the intracellular domain of EGFR and induces EGFR phosphorylation. **(A)** GSEA analysis of genesets for EGFR pathway. NES, normalized enrichment score. Negative NES indicated lower expression in PRDX1-knockout cells. **(B)** Immunoblot assay elucidated that PRDX1 inhibition attenuated EGFR phosphorylation level. **(C,D)** Exogenous Co-IP assay represents that PRDX1 could interact with EGFR in 293T cells. **(E,F)** Endogenous Co-IP assay represents that PRDX1 could interact with EGFR in ACHN and 786-O cells. **(G)** Diagram of EGFR-FL, EGFR-NT, and EGFR-CT. **(H)** Co-IP assay between PRDX1 and EGFR-FL, EGFR-NT, and EGFR-CT in 293-T cell line (VEC = Vector). **(I)** PRDX1 (red) and EGFR (green) co-localization were examined by immunofluorescence assay (scale bars: 10 μm).

**FIGURE 6:**
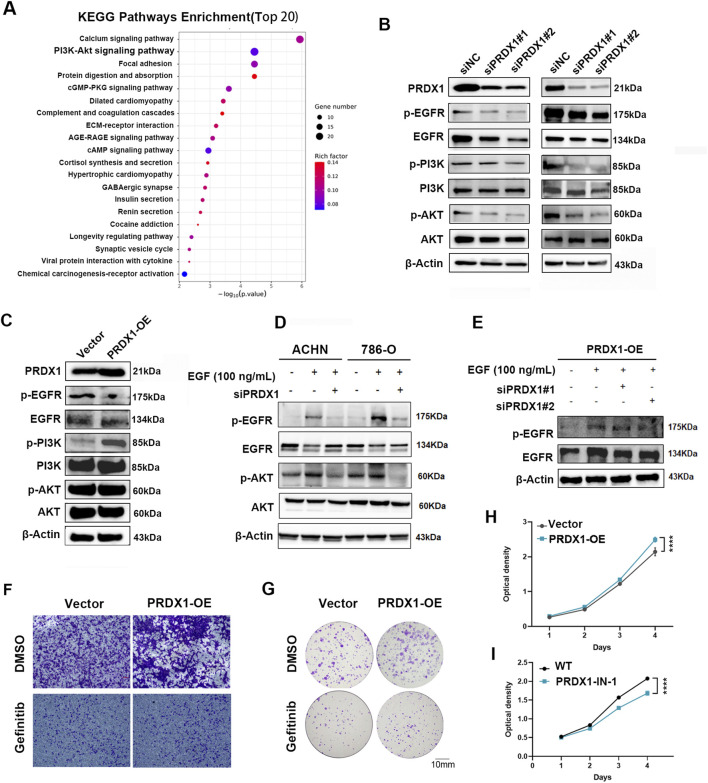
PRDX1 enhances the oncogenic functions via the EGFR/PI3K/AKT signaling axis. **(A)** KEGG analysis showed the PI3K-Akt signaling pathway was significantly downregulated in PRDX1-depleted ACHN cell. Immunoblot assay elucidated that **(B)** PRDX1 inhibition attenuated EGFR/PI3K/AKT phosphorylation level, **(C)** while PRDX1 overexpression exhibited oppositely. **(D)** The inhibition of PRDX1 significantly reduced the EGFR phosphorylation induced by EGF, **(E)** while the overexpression of PRDX1 alleviated this effect. **(F)** Colony formation assays showed the colony number in ACHN cells treated with OE-PRDX1 lentivirus and gefitinib. **(G)** Transwell assay indicated the invasion capacity of ACHN cells treated with OE-PRDX1 lentivirus and gefitinib. CCK-8 assay revealed the viability of ACHN and 786-O cells treated with **(H)** OE-PRDX1 lentivirus and **(I)** PRDX1 inhibitor, PRDX1-IN-1 (n = 6 for independent biological replicates).

To investigate the potential mechanism of PRDX1 in ccRCC malignant progression, GSEA analysis was performed based on the RNA-sequencing data of PRDX1-depleted ACHN cells and control WT cells. Figure 5A implied a lower expression of EGFR pathway in PRDX1-depleted cells. Then, EGF (100 ng/mL) was administered to ccRCC cells to augment EGFR phosphorylation. As shown in Figure 5B, the inhibition of PRDX1 interfered with siRNA significantly reduced the EGFR phosphorylation. Given the central role of EGFR in ccRCC, co-immunoprecipitation (Co-IP) assays was employed to identify the relationship between PRDX1 and EGFR. Firstly, the exogenous Co-IP assay performed using HEK293T cells transfected with Myc-tagged EGFR and HA-tagged PRDX1 alone or in combination, demonstrating that PRDX1 could interact with EGFR in 293T cells (Figures 5C,D). Meanwhile, endogenous PRDX1 could interact with EGFR in both ACHN and 786-O cells (Figures 5E,F). Total EGFR consists of an extracellular (EC) domain, a transmembrane (TM) domain, and an intracellular domain that includes a juxta-membrane (JM) sequence and a kinase domain (Du et al., 2025). For deeply digging the binding-region of EGFR responsible for its interaction with PRDX1, EGFR full length protein (FL), N-terminal intracellular domain that includes a juxta-membrane sequence (NT) as well as C-terminal intracellular region without a juxta-membrane sequence (CT) were constructed for IP assay (Figure 5G). As shown in Figure 6H, PRDX1 interacted with the juxta-membrane (JM) (644–682) domain of EGFR. Moreover, PRDX1 was co-localized with EGFR visualized by immunofluorescence assay (Figure 5I). In summary, these findings presented that PRDX1 interacted with EGFR through its membrane region.

### 3.6 PRDX1 facilitates the oncogenic functions via the EGFR/PI3K/AKT signaling axis

We performed transcriptome sequencing on PRDX1-depleted ACHN cells as well as bioinformatics analysis to reveal the downstream signaling pathway. According to the KEGG analysis, the PI3K-AKT signaling pathway was significantly downregulated (Figure 6A). Further Western blotting experiences verified that the phosphorylation of EGFR, PI3K and AKT was inhibited in PRDX1-depleted ACHN cells, as well as showed oppositely in the ectopic PRDX1 overexpression ACHN cells (Figures 6B,C). Multiple studies report that the PI3K-AKT pathway is a downstream target of EGFR signaling, we presumed that PRDX1 might play an oncogenic role in clear cell RCC via the EGFR/PI3K/AKT signaling axis (Zaryouh et al., 2022). Therefore, EGF (100 ng/mL) was administered to ccRCC cells to stimulate EGFR phosphorylation. As shown in Figures 6D,E, the inhibition of PRDX1 significantly reduced the EGFR phosphorylation induced by EGF, while the overexpression of PRDX1 alleviated this effect. PRDX1-IN-1 (Han et al., 2025), a selective inhibitor of PRDX1, was also employed to verify the inhibitory effect of PRDX1 in ccRCC cells (Figure 6I). Furthermore, to investigate whether PRDX1 inhibitors and EGFR-TKIs can synergistically inhibit the invasion and metastasis of ccRCC cells, gefitinib, a clinical frontline EGFR-TKI for RCC, was co-cultured with overexpressing PRDX1 ccRCC cells. As a result, gefitinib antagonized the effects of overexpressing PRDX1 in ccRCC (Figures 6F–H). In summary, the combination of PRDX1 inhibitors and TKIs has a synergistic effect in RCC treatment.

### 3.7 PRDX1 inhibitor suppresses ccRCC progression via EGFR pathway

**FIGURE 7:**
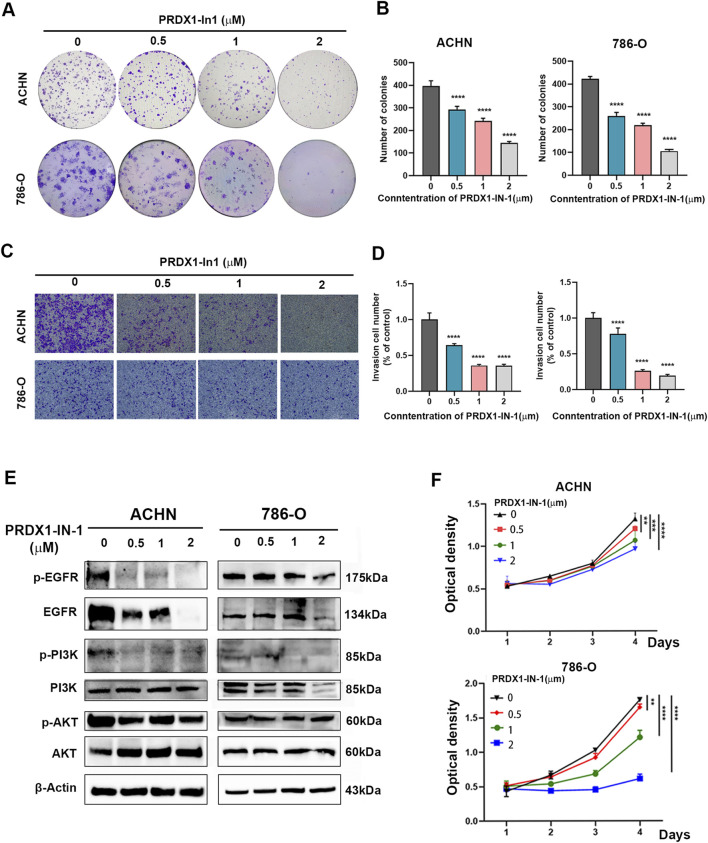
Silencing PRDX1 abolishes the positive effect of EGFR on the proliferation and metastasis of ccRCC cells. **(A,B)** Colony formation assays showed the colony number of ACHN and 786-O cells treated with PRDX1-IN-1 (n = 3 for independent biological replicates). **(C,D)** Transwell assay indicated the invasion capacity of ACHN and 786-O cells treated with PRDX1-IN-1 (n = 3 for independent biological replicates). **(E)** Western blot assay revealed the expression of the indicated proteins in ACHN and 786-O cells treated with PRDX1-IN-1. **(F)** CCK8 assay revealed the viability of ACHN and 786-O cells treated with PRDX1-IN-1 (n = 6 for independent biological replicates).

**FIGURE 8:**
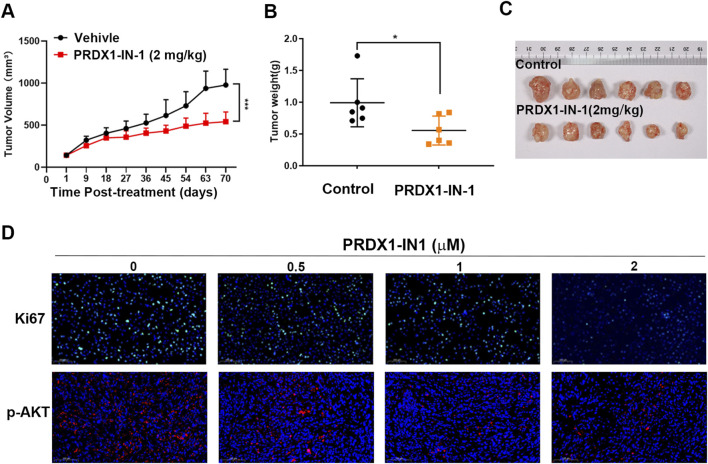
PRDX1-IN-1, a PRDX1 inhibitor, suppresses tumor growth *in vivo*. A dosage of 2 mg/kg PRDX1-IN-1 were administrated into ccRCC tumor bearing nude mice for 10 weeks (n = 6 for each group). The tumor growth curves were documented according to the measurement of **(A,C)** tumor volume, and the **(B)** tumor weight was measured. **(D)** The representative IHC staining of xenograft tumor tissues for Ki67 and p-AKT. Statistical significance was assessed using One-way ANOVA with Dunnett’s multiple comparisons post-test.

Since the potential of PRDX1 in suppressing ccRCC has been discovered, the practical application of PRDX1 inhibitors was investigated in ccRCC. *In vitro*, different concentrations of PRDX1-IN-1 were co-cultured with ACHN and 786-O cells (Figures 7A–F). *In vivo*, PRDX1-IN-1 with 2 mg/kg dosage was administrated in xenograft mouse model (Figures 8A–D). The results showed in Figures 7, 8 indicated that PRDX1-IN-1 significantly reduced the migration, clone formation ability as well as proliferation activity *in vitro* and *in vivo*.

## 4 Discussion

The alteration of redox state is a common characteristic of cancer, as the dysregulation of cellular metabolism and the abnormal activation of signal transduction triggered by various genetic variations often leads to an increase in the production of reactive oxygen species (ROS) within the cells (Inigo et al., 2021). PRDX1 is a member of the typical 2-Cys PRDX which belongs to the peroxiredoxin protein family (Lee, 2020). As the most abundant and ubiquitously distributed PRDX isoform, PRDX1 is crucial in maintaining a correct redox balance to ensure cell survival (Neumann et al., 2003). In recent years, plenty of studies have shown that PRDX1 was a potential anti-tumor target in various cancers (Wieduwilt and Moasser, 2008; Xu et al., 2023; Jiang et al., 2025; Kiran et al., 2025; Motzer et al., 2025). However, the role of PRDX1 in RCC still remains unclear. Although our previous study predicted that PRDX1 might be a potential prognostic factor in RCC (Zhou et al., 2019), further experimental validations were still lacking. This research could bridge this blank gap and elucidate the mechanism of PRDX1 in the development and progression of RCC.

In this research, we utilized the ULACAN and GEO databases combined with tissue microarrays (TMAs) of human ccRCC to evaluate the expression of PRDX1 in various stages of ccRCC and the correlation with clinical factors and survival outcomes. Age, extent of lymph node metastases (N), occurrence of distant metastases (M), grade and the TNM system were found to be independent risk factors for tumor progression. PRDX1 expression in patients with early-stage renal cancer was significantly lower than in those with advanced-stage disease. These data shown that the expression of PRDX1 is strongly associated with tumor cell proliferation and growth. Both *in vitro* and *in vivo* experiments proved the oncogenic role of PRDX1 in ccRCC.

It is well-known that epithelial-mesenchymal transition (EMT), characterized by progressive loss of epithelial traits and acquisition of mesenchymal phenotype (Ding et al., 2021), plays a crucial role in tumor progression. Although the significance of PRDX1 is known to be involved in tumor progression, including invasion and metastasis, its role in EMT during tumorigenesis still remains unclear. Bin Ha et al. revealed that overexpression of PRDX1 could enhance TGF-β1-induced epithelial-to-mesenchymal transition (EMT) and cell migration in A549 cells, while PRDX1 knockdown could block EMT and cell migration (Ha et al., 2012). In this study, we examined the expression levels of two EMT markers, E-cadherin and N-cadherin, as well as the key EMT transcription factor Snail in ACHN cell line. It’s observed that the downregulation of PRDX1 reduced the key EMT transcription factor Snail, triggering the decrease of the E-cadherin expression and the increase of the N-cadherin expression, indicating that PRDX1 is an inducer of EMT in ACHN cell.

Previous researches revealed that PI3K could mediate EMT via the activation of AKT (Shih et al., 2012). By transcriptome analysis, we found that PRDX1 regulated the EGFR tyrosine kinase inhibitors resistance pathway and PI3K/AKT signalling pathway. In addition, silencing PRDX1 could inhibit the phosphorylation of EGFR, PI3K, and AKT. These data suggest that PRDX1 may regulate EMT process in ACHN cells through regulating EGFR/PI3K/AKT signalling.

Epidermal growth factor receptor (epidermal growth factor receptor, EGFR) has been proven to be overexpressed in renal cancer, which may be related to the insensitivity of RCC towards radiotherapy and chemotherapy. Studies found that the most common mutations are in exon 19 (45%) and exon 21 (40%–45%), which account for approximately 85%–90% of all EGFR mutations. These mutations occur more frequently, and is sensitive to targeted therapies, such as EGFR-TKIs (Schenk et al., 2021). Advanced targeted drugs with new mechanism are urgently needed to overcome the drug resistance caused by EGFR mutations. There is no doubt that finding new therapeutic drugs will require a very long time since the resistance becoming increasingly stronger.

In this study, we discovered that the phosphorylation of EGFR was inactivated caused by siPRDX1 transfection and PRDX1 inhibitor, PRDX1-IN-1. A series of biochemical experiments indicated that PRDX1 interacted with the juxta-membrane (JM) domain of EGFR, which has been proven crucial for EGFR phosphorylation (Thiel and Carpenter, 2007). Besides, we discovered that the binding site of PRDX1-EGFR (644–682) is not located in the mutation sites (exon 19 and exon 21) which account for nearly 90% of mutations. Taking together, our findings of this study shed light on future research about the novel molecular mechanism of PRDX1 in ccRCC and support that PRDX1 might potentially be a therapeutic target, even for patients resistant to EGFR-TKIs. The future works on PRDX1 inhibitors may act as a therapeutic candidate for treatment of renal cancer.

## Data Availability

The datasets presented in this study can be found in online repositories. The names of the repository/repositories and accession number(s) can be found in the article/Supplementary Material.
