# Supplementary material for: PRDX1 promotes clear cell renal cell carcinoma progression by modulating EGFR-dependent AKT pathway activation
Source: Front Pharmacol. 2026 Apr 17;17:1741879. doi: 10.3389/fphar.2026.1741879 (PMC13132848; doi:10.3389/fphar.2026.1741879)
Supplement: Supplementary file 1 [file Supplementaryfile1.docx]

Supplementary Material

#
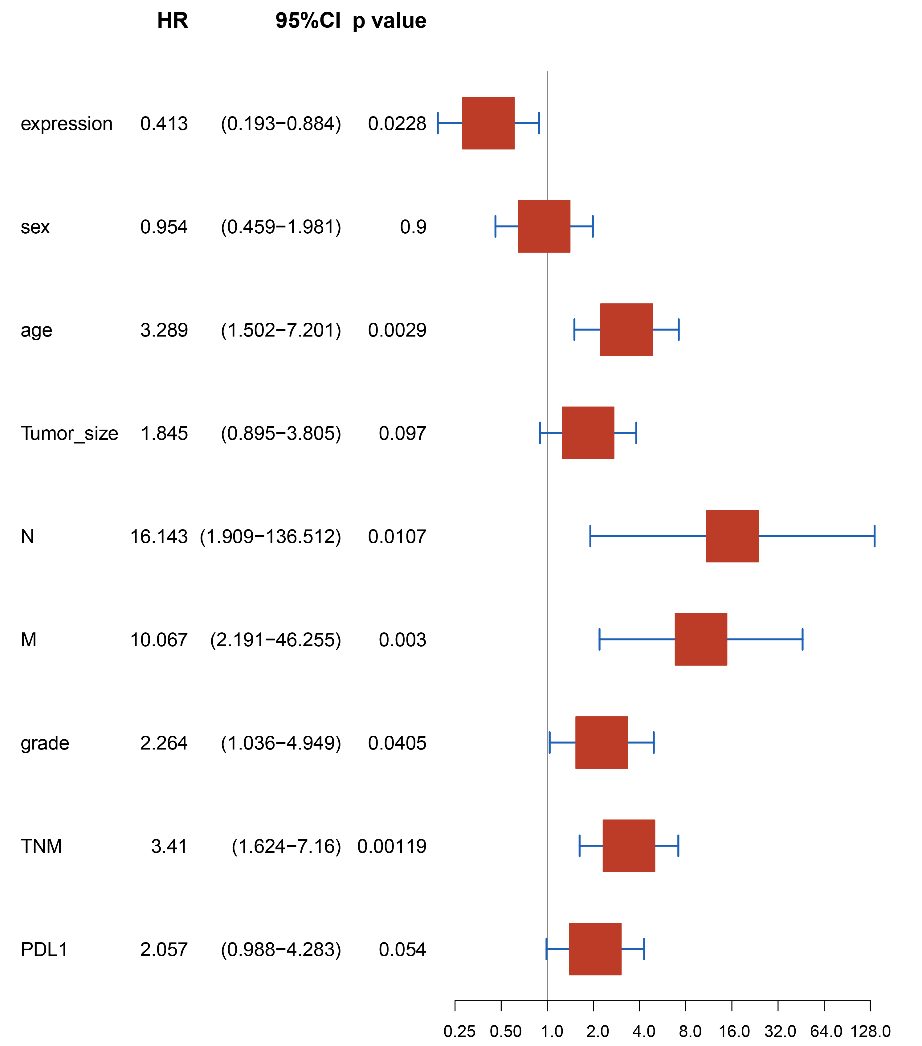
1 Supplementary Figures

**Supplementary Figure 1.** Univariate Cox proportional-hazard model was used to identify independent prognostic factors for ccRCC patients. Baseline characteristics of 90 pairs of ccRCC samples on overall survival. Age, extent of lymph node metastases (N), occurrence of distant metastases (M), grade and the TNM system were found to be risk factors for tumor progression. HR=hazard ratio. 95%Cl= 95% Confidence Level.


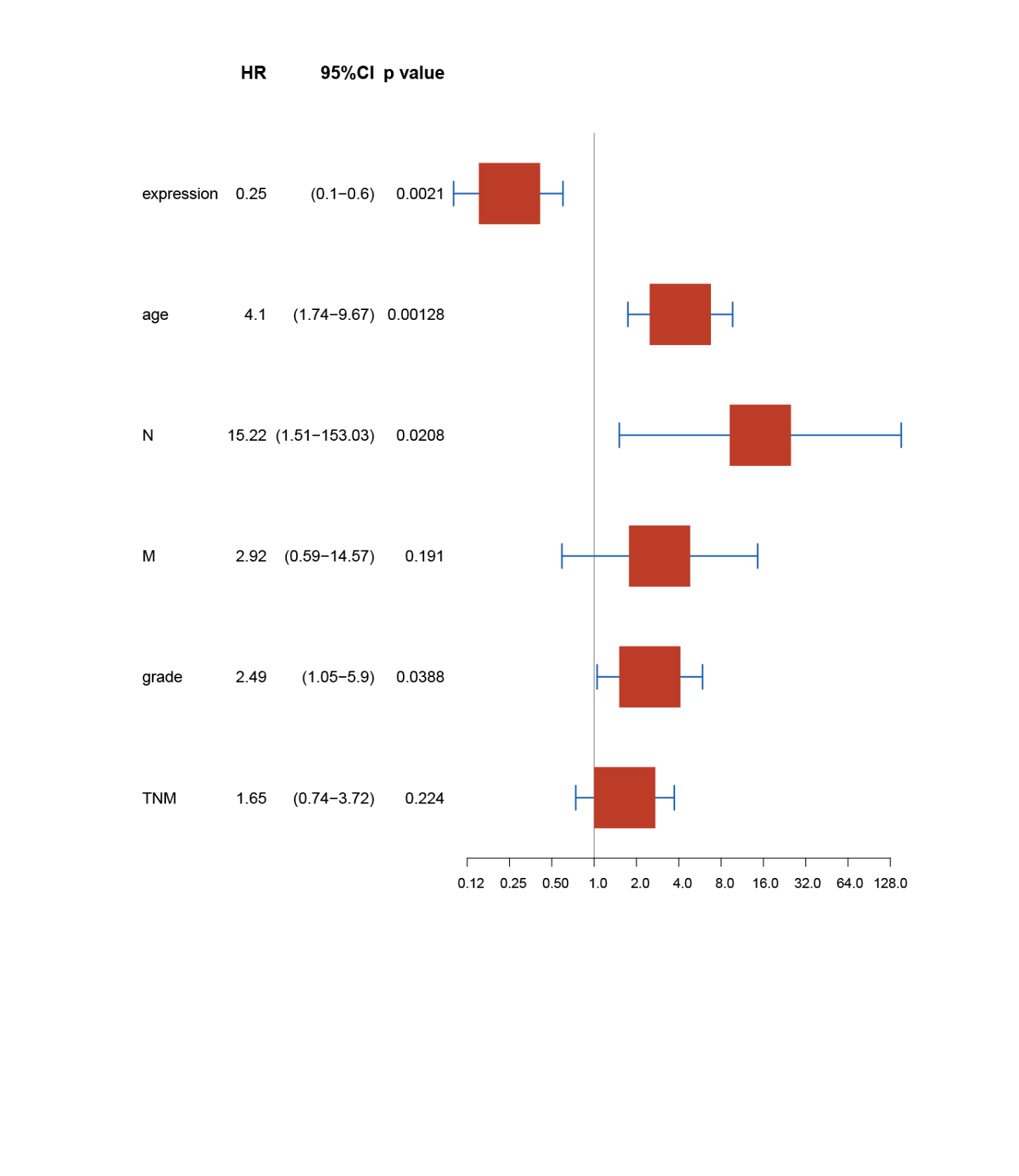


**Supplementary Figure 2.** Multivariable logistic regression analyses were performed to identify independent predictors for ccRCC patients. Baseline characteristics of 90 pairs of ccRCC samples on overall survival. Age, extent of lymph node metastases (N), occurrence of distant metastases (M), grade and the TNM system were found to be independent risk factors for tumor progression. HR=hazard ratio. 95%Cl= 95% Confidence Level.

**2 Supplementary Tables**

**Table S1**: List of sequences used in this study.

| **Gene** | **sense** | **antisense** |
| --- | --- | --- |
| PRDX1 | TCCTTTGGTATCAGACCCGA | TAAAAAGGCCCCTGAACGAG |
| β-Actin | ACGTGGACATCCGCAAAGAC | CAAGAAAGGGTGTAACGCAACTA |
| PRDX1-siRNA1 | GAUGAGACUUUGAGACUAGUUTT | AACUAGUCUCAAAGUCUCAUCTT |
| PRDX1-siRNA2 | CCAGAUGGUCAGUUUAAAGAUTT | AUCUUUAAACUGACCAUCUGGTT |
| sgPRDX1(Crispr-Cas9) | ACTGAAAGCAATGATCTCCGTGG | |

**Table S2**: Cox multivariate regression analysis of baseline characteristics of ccRCC samples.

|  | **Univariate analysis(95%CI)** | | | | **Multivariate analysis(95%CI)** | | | | |
| --- | --- | --- | --- | --- | --- | --- | --- | --- | --- |
| **variables** | **HR** | **lower-bound** | **Upper-bound** | **p-value** | **HR** | **lower-bound** | **Upper-bound** | **p-value** |  |
| **expression** | 0.413 | 0.193 | 0.884 | 0.0228 | 0.25 | 0.1 | 0.6 | 0.0021 |  |
| **sex** | 0.954 | 0.459 | 1.981 | 0.9 |  |  |  |  |  |
| **age** | 3.289 | 1.502 | 7.201 | 0.0029 | 4.1 | 1.74 | 9.67 | 0.00128 |  |
| **Tumor size** | 1.845 | 0.895 | 3.805 | 0.097 |  |  |  |  |  |
| **N** | 16.143 | 1.909 | 136.512 | 0.0107 | 15.22 | 1.51 | 153.03 | 0.0208 |  |
| **M** | 10.067 | 2.191 | 46.255 | 0.003 | 2.92 | 0.59 | 14.57 | 0.191 |  |
| **grade** | 2.264 | 1.036 | 4.949 | 0.0405 | 2.49 | 1.05 | 5.9 | 0.0388 |  |
| **TNM** | 3.41 | 1.624 | 7.16 | 0.00119 | 1.65 | 0.74 | 3.72 | 0.224 |  |
| **PDL1** | 2.057 | 0.988 | 4.283 | 0.054 |  |  |  |  |  |

**Table S3:** Actual P value shown in the figures were listed below:

| **Figure 1B (left above): PRDX1 expression in KIRC** | |
| --- | --- |
| Comparison | Statistical significance |
| Normal-vs-N0 | 1.62447832963153E-12**** |
| Normal-vs-N1 | 8.391300E-04*** |
| N0-vs-N1 | 3.275800E-02* |
| **Figure 1B (right above): PRDX1 expression in KIRC** | |
| Comparison | Statistical significance |
| Grade 1-vs-Grade 2 | 1.118410E-01 |
| Grade 1-vs-Grade 3 | 5.778100E-02 |
| Grade 1-vs-Grade 4 | 1.657120E-03** |
| Grade 2-vs-Grade 3 | 5.217800E-01 |
| Grade 2-vs-Grade 4 | 1.120460E-02* |
| Grade 3-vs-Grade 4 | 3.543600E-02* |
| **Figure 1B (left below): GSE73731 cohort** | |
| Comparison | Statistical significance |
| Grade 4-vs-Grade 1 | 0.0248* |
| Grade4-vs-Grade 2 | 0.0044** |
| Grade4-vs-Grade 3 | 0.0033** |
| **Figure 1B (right below): GSE53757 cohort** | |
| Comparison | Statistical significance |
| Grade 4-vs-Grade 1 | 0.0097** |
| Grade 4-vs-Grade 2 | 0.2968 |
| Grade 4-vs-Grade 3 | 0.9667 |
| **Figure 2A: Relative PRDX1 mRNA** | |
| Comparison | Statistical significance |
| ACHN siNC vs. siPRDX1#1 | 0.0092** |
| ACHN siNC vs. siPRDX1#2 | 0.0144* |
| 786-O siNC vs. siPRDX1#1 | <0.0001**** |
| 786-O siNC vs. siPRDX1#2 | <0.0001**** |
| **Figure 2C: Optical density** | |
| Comparison | Statistical significance |
| ACHN siNC vs. siPRDX1#1 | <0.0001**** |
| ACHN siNC vs. siPRDX1#2 | <0.0001**** |
| 786-O siNC vs. siPRDX1#1 | 0.0092** |
| 786-O siNC vs. siPRDX1#2 | 0.0144* |
| **Figure 2E: Number of clonies** | |
| Comparison | Statistical significance |
| ACHN siNC vs. siPRDX1#1 | 0.00017*** |
| ACHN siNC vs. siPRDX1#2 | 0.00075*** |
| 786-O siNC vs. siPRDX1#1 | 0.00033*** |
| 786-O siNC vs. siPRDX1#2 | 0.00127** |
| **Figure 2I: Relative migration ability (%)** | |
| Comparison | Statistical significance |
| ACHN siNC vs. siPRDX1#1 | 0.0025** |
| ACHN siNC vs. siPRDX1#2 | 0.0011** |
| 786-O siNC vs. siPRDX1#1 | 0.0072** |
| 786-O siNC vs. siPRDX1#2 | 0.0043** |
| **Figure 3B: Relative PRDX1 mRNA level (PRDX1/β-Actin)** | |
| Comparison | Statistical significance |
| ACHN shNC vs. shPRDX1-1 | 0.0106* |
| ACHN shNC vs. shPRDX1-2 | 0.0419* |
| **Figure 3C: Tumor weight** | |
| Comparison | Statistical significance |
| ACHN shNC vs. shPRDX1-1 | <0.0001**** |
| ACHN shNC vs. shPRDX1-2 | <0.0001**** |
| **Figure 3E: Tumor volume** | |
| Comparison | Statistical significance |
| ACHN shNC vs. shPRDX1-1 | 0.00039*** |
| ACHN shNC vs. shPRDX1-2 | 0.00034*** |
| **Figure 6H&I: Optical density** | |
| Comparison | Statistical significance |
| ACHN Vector vs. PRDX1-OE | <0.0001**** |
| ACHN WT vs. PRDX1-IN-1 | <0.0001**** |
| **Figure 7B: Number of colonies** | |
| Comparison (μM) for ACHN | Statistical significance |
| 0 vs. 0.5 | <0.0001**** |
| 0 vs. 1 | <0.0001**** |
| 0 vs. 2 | <0.0001**** |
| Comparison (μM) for 786-O |  |
| 0 vs. 0.5 | <0.0001**** |
| 0 vs. 1 | <0.0001**** |
| 0 vs. 2 | <0.0001**** |
| **Figure 7D: Invasion cell number (% of control)** | |
| Comparison (μM) for ACHN | Statistical significance |
| 0 vs. 0.5 | <0.0001**** |
| 0 vs. 1 | <0.0001**** |
| 0 vs. 2 | <0.0001**** |
| Comparison (μM) for 786-O |  |
| 0 vs. 0.5 | <0.0001**** |
| 0 vs. 1 | <0.0001**** |
| 0 vs. 2 | <0.0001**** |
| **Figure 7F: CCK8 assay** | |
| Comparison (μM) for ACHN | Statistical significance |
| 0 vs. 0.5 | 0.0041** |
| 0 vs. 1 | 0.0007*** |
| 0 vs. 2 | <0.0001**** |
| Comparison (μM) for 786-O | Statistical significance |
| 0 vs. 0.5 | 0.0079** |
| 0 vs. 1 | <0.0001**** |
| 0 vs. 2 | <0.0001**** |
